# Comparison of Estimated Effectiveness of Case-Based and Population-Based Interventions on COVID-19 Containment in Taiwan

**DOI:** 10.1001/jamainternmed.2021.1644

**Published:** 2021-04-06

**Authors:** Ta-Chou Ng, Hao-Yuan Cheng, Hsiao-Han Chang, Cheng-Chieh Liu, Chih-Chi Yang, Shu-Wan Jian, Ding-Ping Liu, Ted Cohen, Hsien-Ho Lin

**Affiliations:** 1 Institute of Epidemiology and Preventive Medicine, National Taiwan University College of Public Health, Taipei, Taiwan; 2 Epidemic Intelligence Center, Taiwan Centers for Disease Control, Taipei, Taiwan; 3 Department of Pediatrics, National Taiwan University Children’s Hospital, Taipei, Taiwan; 4 Institute of Bioinformatics and Structural Biology, National Tsing Hua University, Hsinchu City, Taipei; 5 National Taipei University of Nursing and Health Sciences, Taipei, Taiwan; 6 Department of Epidemiology of Microbial Diseases and the Public Health Modelling Unit, Yale School of Public Health, New Haven, Connecticut; 7 Global Health Program, National Taiwan University College of Public Health, Taipei, Taiwan

## Abstract

**Importance:**

Taiwan is one of the few countries with initial success in COVID-19 control without strict lockdown or school closure. The reasons remain to be fully elucidated.

**Objective:**

To compare and evaluate the effectiveness of case-based (including contact tracing and quarantine) and population-based (including social distancing and facial masking) interventions for COVID-19 in Taiwan.

**Design, Setting, and Participants:**

This comparative effectiveness study used a stochastic branching process model using COVID-19 epidemic data

from Taiwan, an island nation of 23.6 million people, with no locally acquired cases of COVID-19 reported for 253 days between April and December 2020.

**Main Outcomes and Measures:**

Effective reproduction number of COVID-19 cases (the number of secondary cases generated by 1 primary case) and the probability of outbreak extinction (0 new cases within 20 generations). For model development and calibration, an estimation of the incubation period (interval from exposure to symptom onset), serial interval (time between symptom onset in an infector-infectee pair), and the statistical distribution of the number of any subsequent infections generated by 1 primary case was calculated.

**Results:**

This study analyzed data from 158 confirmed COVID-19 cases (median age, 45 years; interquartile range, 25-55 years; 84 men [53%]). An estimated 55% (95% credible interval [CrI], 41%-68%) of transmission events occurred during the presymptomatic stage. In our estimated analysis, case detection, contact tracing, and 14-day quarantine of close contacts (regardless of symptoms) was estimated to decrease the reproduction number from the counterfactual value of 2.50 to 1.53 (95% CrI, 1.50-1.57), which would not be sufficient for epidemic control, which requires a value of less than 1. In our estimated analysis, voluntary population-based interventions, if used alone, were estimated to have reduced the reproduction number to 1.30 (95% CrI, 1.03-1.58). Combined case-based and population-based interventions were estimated to reduce the reproduction number to below unity (0.85; 95% CrI, 0.78-0.89). Results were similar for additional analyses with influenza data and sensitivity analyses.

**Conclusions and Relevance:**

In this comparative effectiveness research study, the combination of case-based and population-based interventions (with wide adherence) may explain the success of COVID-19 control in Taiwan in 2020. Either category of interventions alone would have been insufficient, even in a country with an effective public health system and comprehensive contact tracing program. Mitigating the COVID-19 pandemic requires the collaborative effort of public health professionals and the general public.

## Key Points

**Question:**

What are the explanations for the initial success of COVID-19 control in Taiwan, a country that has one of the lowest per capita incidence and mortality rates in the world?

**Findings:**

In this comparative effectiveness research study that used detailed epidemiologic and contact tracing data, neither case-based interventions (including contact tracing and quarantine) or population-based interventions (including social distancing and facial masking) alone were estimated to have been sufficient to contain COVID-19. The combination of case-based and population-based interventions was needed.

**Meaning:**

The combination of case-based interventions with population-based interventions with wide adherence may explain the success of COVID-19 control in Taiwan.

## Teaser

This comparative effectiveness research study examines case-based and population-based interventions and their association with COVID-19 control in Taiwan.

## Introduction

During the COVID-19 pandemic, some countries successfully contained the first wave of the outbreak with strong nonpharmaceutical interventions, such as strict lockdowns and border closures.^1,2,3,4,5,6,7^ Taiwan, an island nation of 23.6 million people, was initially considered a high-risk country for COVID-19, given its close geographic and economic relationships with China. Nevertheless, almost 1 year after the outbreak, Taiwan had one of the lowest per capita incidence and mortality rates of COVID-19 in the world. During a 253-day period from early April until December 2020, no cases originating in Taiwan were confirmed.^8^ As of February 28, 2021, there had been 955 confirmed cases of COVID-19 in Taiwan, of which only 77 (8.1%) were locally acquired. Notably, the containment of COVID-19 was achieved without strict lockdown or school closure.^9^

To prevent the health care system from being overwhelmed, Taiwan implemented a “containment-as-mitigation,” or elimination, strategy.^6,10,11^ This approach included border control, case-based interventions for COVID-19 patients, and population-based measures for the general public.^9,10,11,12^ Border control was an essential tool to buy time for better preparation and limit the number of imported cases from other countries. This was accompanied by interventions to contain local transmissions that resulted from importation events. The case-based interventions included case detection and isolation through sensitive surveillance systems, contact tracing of confirmed cases to facilitate early detection of secondary cases among close contacts, and 14-day quarantine of close contacts (regardless of symptoms). The population-based measures included use of face masks, personal hygiene, and physical distancing.^9,11,12,13^ Although most population-based measures were recommended by the Central Epidemic Command Center during the early phase of the epidemic, some became compulsory as the pandemic and local epidemic developed (eg, mandatory face mask use on public transportations after April 2020).^9^

Despite Taiwan’s initial success against COVID-19, it remains unclear which interventions contributed substantially to containment, as the various measures were implemented simultaneously from the beginning of the pandemic. Although vaccination programs are being rapidly implemented in many countries, a better understanding of the potential effects of nonpharmaceutical interventions and optimizing their use in different settings is essential before herd immunity is achieved.^1,2,3,14^ Several modeling studies have examined the effectiveness of case-based or population-based interventions. However, most studies simulated hypothetical scenarios without empirical links to specific real-world settings and primary data.^14,15,16,17,18^ We combined transmission modeling and detailed epidemiologic and contact tracing data to estimate the effectiveness of different COVID-19 interventions in Taiwan. Our analysis focused on local transmission after the introduction of imported cases, not on border control.

## Methods

### Study Design

We sought to quantify the effects of case-based and population-based interventions by estimating the effective reproduction number (the number of secondary cases generated by one primary case) under case-based interventions (R_c), population-based interventions (R_p), and both (R_pc). The conceptual framework is depicted in eFigure 1 in the Supplement. Applying a calibrated transmission model that incorporated the natural history of COVID-19 and the process of care seeking, isolation, and quarantine based on case data, we first estimated the effective reproduction number R_c when the input basic reproduction number (R_0_, without any interventions) ranged between 2 and 3. Second, we reran the transmission model separately and fit the model to the cluster size of transmission chains of COVID-19 cases in Taiwan to jointly estimate R_p and R_pc. Details are described later in the article and in the eMethods in the Supplement. The study data were collected as part of the outbreak response and surveillance by the pronouncement of the Central Epidemic Control Center, which was established in accordance with Article 17 of the Communicable Disease Control Act in Taiwan.^19^ The Taiwan Centers for Disease Control (CDC) approved the study and waived institutional review board approval and informed consent, and all data were deidentified before analysis.

### Data

Case series data of SARS-CoV-2 infections in Taiwan were collected from the official website of Taiwan CDC^20^ and reviewed by Taiwan CDC officers to clarify missing information. All cases were confirmed by reverse transcription–polymerase chain reaction testing.^8^ Cases were isolated immediately after being notified to the Taiwan CDC. We analyzed the epidemiological and contact tracing data to characterize the transmission dynamics of COVID-19.^21^ Starting on March 21, 2020, all inbound passengers (citizens and eligible noncitizens) to Taiwan were required to undergo a 14-day quarantine on entry; nearly all confirmed cases after this date and before February 28, 2021 (742 of 786 cases [94.4%]) were imported and were mostly diagnosed during or soon after the quarantine.^8,9^ Therefore, we included locally acquired cases, epidemiologically confirmed clusters, and imported cases in people who entered Taiwan before March 21, 2020, in the analysis of case series data. We excluded people who were returning to Taiwan and tested at the airport or who received a diagnosis during home quarantine.

### Transmission Model

We adapted the stochastic branching process model developed by Hellewell et al^17^ to explicitly incorporate case-based interventions. The model generated transmission trees by drawing the number of secondary cases for each primary case based on the statistical distribution of the reproduction number. For each expected pair of primary-secondary cases, the incubation period, onset-to-isolation interval, and generation interval (time between infection events in an infector-infectee pair) were determined by statistical sampling from the estimated distributions. Transmission would occur if the sampled generation interval was outside the isolation or quarantine period of the primary case, whereas transmission would be prevented if the sample generation interval was inside the isolation or quarantine period (eFigure 2 in the Supplement). Per current policy in Taiwan, we assumed in this model that testing and isolation occurred at the same time.^12,21^ Parameter values of the transmission model were estimated from empirical case data or extracted based on literature review (Table^7,8,12,22,23,24^). We assumed that 40% of incident cases were asymptomatic and were 50% less transmissible than symptomatic cases.^22,25^ Presymptomatic infection was assumed to be as transmissible as symptomatic infection.^21^ The proportion of presymptomatic transmission and the standard deviation of the generation interval were estimated by fitting the transmission model to the observed serial intervals (time between symptom onset in an infector-infectee pair) using the Sequential Monte Carlo algorithm.^15,26^ A 1-way sensitivity analysis was conducted to evaluate the association of parameter values with the projected R_c (see eMethods and eTables 1-2 in the Supplement for details of the transmission model).

**Table.** Parameters for the Branching Process Model

Fixed parameter	Point estimate	Range for 1-way sensitivity analysis	Source/notes
Incubation period, mean (SD), d	5.50 (3.26)	1.06 to 13.45	Estimated directly from data, gamma distributed (eMethods in the Supplement)
Onset-to-isolation interval, mean (SD), d	5.02 (5.80)	−0.81 to 20.51	
Basic reproduction No. (R_0_)	2.5	2.0 to 3.0	Extracted from literature^7,22,23,24^
Probability of being asymptomatic	0.4	0.20 to 0.60	
Relative transmissibility of asymptomatic case	0.5	0 to 1	
Probability of case detection	0.95	0.75 to 1	Assumed based on local data and previous reports^8,12^
Probability of contact ascertainment	0.9	0.75 to 1	Assumed based on local data and previous reports^8,12^
Duration of quarantine, d	14		Local policy
Backtracking days for quarantined contacts, d	4		
**Fitted parameter**	**Prior**	**Posterior estimate (95% CrI)**	
Probability of presymptomatic transmission	Uniform (0.01 to 0.99)	0.55 (0.41 to 0.68)	Fitted to the observed serial interval (eMethods and eFigure 3 in the Supplement)
Standard deviation of the generation interval, d	Uniform (0.001 to 5)	2.70 (1.88 to 3.76)	

Abbreviation: CrI, credible interval.

### Estimating the Effectiveness of Case-Based and Population-Based Interventions

We estimated the effectiveness of case-based interventions using the calibrated transmission model. The input reproduction number of the transmission model (eFigure 1 in the Supplement), the counterfactual R_0_ (the hypothetical reproduction number without interventions), was assumed to be 2.50 (range, 2-3), which was similar to the estimated R_0_ in Hong Kong at the beginning of its outbreak and consistent with the previously estimated R_0_ values.^7,23,24^ The effective reproduction numbers under 5 scenarios of case-based interventions were considered (eTable 3 in the Supplement): (1) no case-based interventions; (2) case detection and isolation; (3) case detection and isolation, and contact tracing to detect and isolate secondary cases; (4) case detection and isolation, contact tracing, and 7-day quarantine for contacts regardless of symptoms; and (5) case detection and isolation, contact tracing, and 14-day quarantine (current policy). The primary indicator was the mean effective reproduction number, along with the probability of outbreak extinction, which was defined as 0 new cases within 20 generations.

The effective reproduction numbers R_p and R_pc were estimated by fitting the transmission model to the observed size of transmission clusters in Taiwan (eFigure 1 in the Supplement). We reran the calibrated transmission model, setting the input reproduction number to an unknown parameter R_p (assuming a wide uniform distribution) to represent the scenario in which population-based interventions were already in place. The corresponding output reproduction number from this model (incorporating case-based interventions) would be R_pc, representing the scenario of joint case-based and population-based interventions (the actual situation in Taiwan). Using the Sequential Monte Carlo algorithm, the transmission model was fit to the size distribution of the self-limited transmission chains that were observed in Taiwan to estimate the prior parameter R_p and the model output R_pc.

### Additional Analyses for R_p and R_pc

We conducted additional analyses on R_p and R_pc using different methods and sources of information to cross-check the estimates from the transmission model. First, we estimated the time-varying reproduction number (Rt) of seasonal influenza before and after the COVID-19 outbreak, because the population-based interventions would likely have an association with other respiratory infections (eMethods in the Supplement).^7,27^ The Rt of influenza was estimated using the time-series data of influenza cases with severe complications (a notifiable condition in Taiwan), the frequency of consulting physicians about influenza-like illness, and the proportion of influenza-positive specimens among the samples of patients with respiratory infection.^28,29^ Second, we analytically estimated the effective R_pc using the observed size of transmission clusters.^30^ The point estimate of R_pc was estimated using the formula *R = 1-1/m*, in which m was the average size of clusters, and the 95% CI of R_pc was estimated using bootstrapping. All statistical analyses were conducted in R, version 3.6.3, and RStan, version 2.19.3 (R Foundation).

## Results

### Epidemiology and Transmission Dynamics of COVID-19 in Taiwan

**Figure 1.**
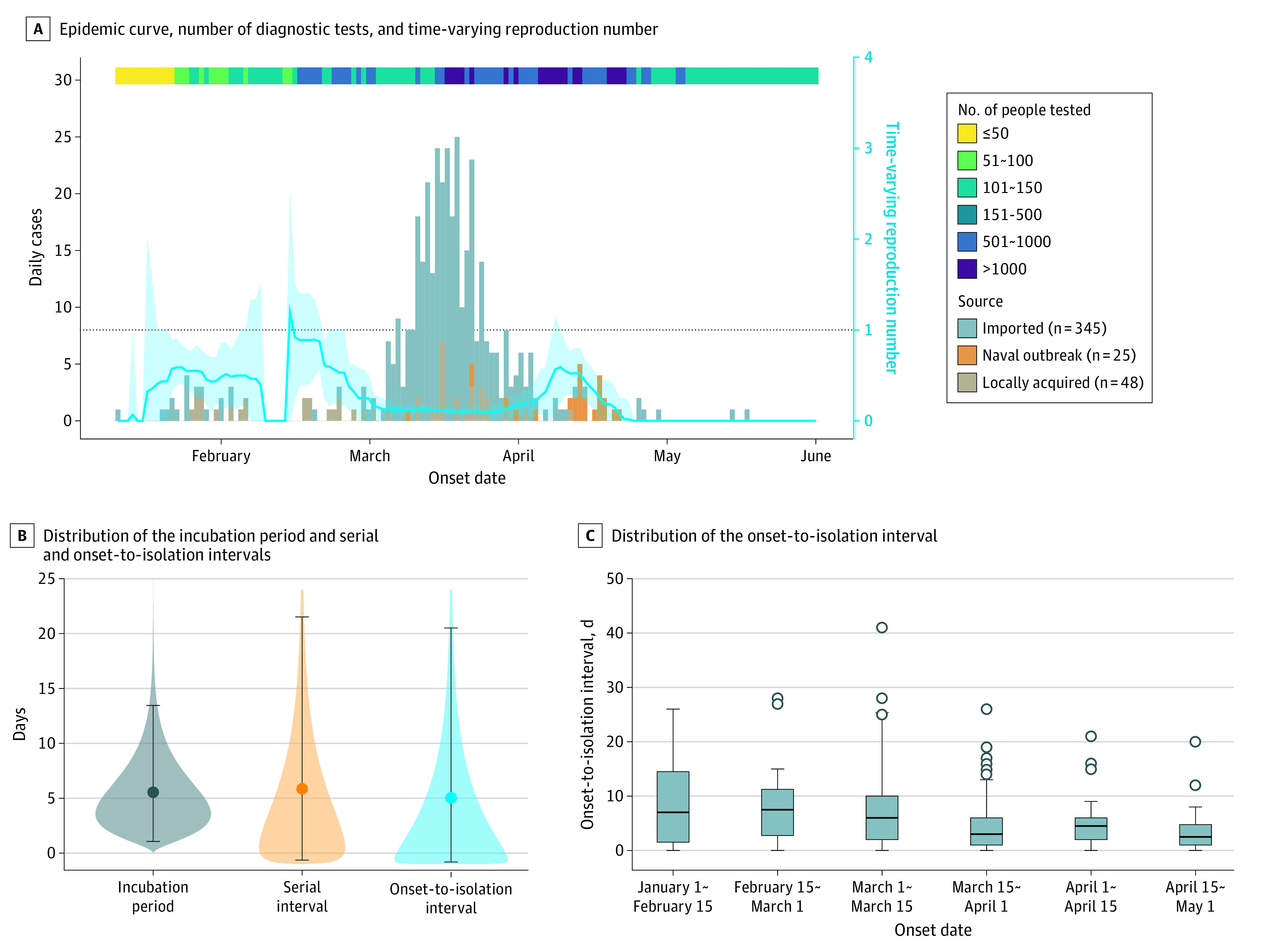
Epidemiological Characteristics and Parameters of the COVID-19 Cases in Taiwan From January 10 to June 1, 2020 A, The epidemic curve, number of diagnostic tests by day, and the time-varying reproduction number. The blue line and the light blue shading represent the point estimate and 95% confidence interval of time-varying reproduction number of COVID-19. B, Distribution of the incubation period, serial interval, and onset-to-isolation interval. The points and the intervals represent the mean estimates and the 2.5 and 97.5 percentiles of the estimated distribution. The shaded areas represent the mean estimation of the interval distribution. C, Distribution of the onset-to-isolation interval by onset date. The central lines indicate the median, boxes indicate interquartile ranges, whiskers indicate the upper and lower adjacent values (within 1.5-fold of the interquartile range), and isolated points indicate outliers.

The COVID-19 epidemic in Taiwan started with a few imported cases from China, followed by nonsustained local transmission during January and February 2020 (Figure 1A). In March, a surge of imported cases, mainly from North America and Europe, was followed by sporadic local transmission. The criteria of notification and testing were gradually expanded, with an overall positive test rate of 0.61% (Figure 1A).^10^ Case series data from 158 confirmed COVID-19 cases (median age, 45 years; interquartile range, 25-55 years; 84 men [53%]) were analyzed to estimate the incubation period, onset-to-isolation interval, and serial interval. The estimated mean incubation period and mean serial interval were 5.50 (95% credible interval [CrI], 1.06-13.45) and 5.86 (95% CrI, −0.64 to 21.51) days, respectively (Figure 1B). The mean onset-to-isolation interval was 5.02 (95% CrI, −0.81 to 20.51) days, with a decreasing trend over time (Figure 1C). By fitting the transmission model to the observed serial intervals, we estimated that 55% (95% CrI, 41%-68%) of transmission events occurred during the presymptomatic stage.

### Effectiveness of Case-Based Interventions

**Figure 2.**
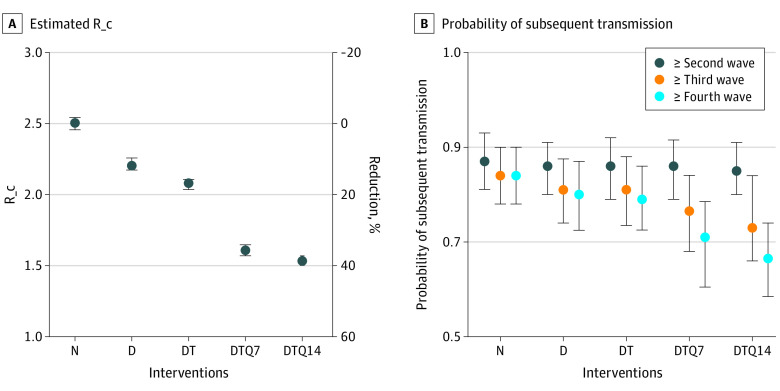
Effective Reproduction Number (R_c) of COVID-19 Cases Under Different Combination of Case-Based Interventions Based on the Fitted Transmission Model Results were based on 1000 stochastic simulations under the counterfactual R_0_ value of 2.5 and 100 introductions. Whiskers indicate 95% credible intervals. A, Estimated R_c and the proportional reduction of R_c from the counterfactual value of 2.5. B, The probability of subsequent transmission from an index case. D indicates case detection; N, none of the case-based interventions are implemented; T, contact tracing; Q7/Q14, quarantine of contacts for 7 or 14 days.

Using the fitted transmission model, we found that the combination of case detection, contact tracing, and 14-day quarantine of close contacts (regardless of symptoms) could lower the R_c from the counterfactual value of 2.50 (R_0_) to 1.53 (95% CrI, 1.50-1.57), or a 39% reduction (Figure 2A). With 100 initial cases introduced to the community (ie, cases escaping the attention of border control), the estimated probability of epidemic extinction was 0% (95% CrI, 0%-0%). In the 1-way sensitivity analysis, the most significant parameter for R_c was the onset-to-isolation interval, followed by the incubation period, the counterfactual R_0_, and the relative transmissibility of asymptomatic cases (eFigure 3 in the Supplement). Notably, the projected R_c would always be more than 1 when the input counterfactual R_0_ was set at 2 to 3. Among different case-based interventions, quarantine of contacts contributed the most to the reduction of R_c (Figure 2A). Case-based interventions could not substantially prevent the secondary transmission of cases, but could still reduce tertiary transmissions that resulted from secondary cases and quaternary transmissions from tertiary cases if the close contacts could be quarantined (Figure 2B). We found that reducing the duration of quarantine from 14 days to 7 days would only slightly increase the R_c from 1.53 (95% CrI, 1.50-1.57) to 1.61 (95% CrI, 1.57-1.65) (Figure 2A).

### Effectiveness of Population-Based Interventions

**Figure 3.**
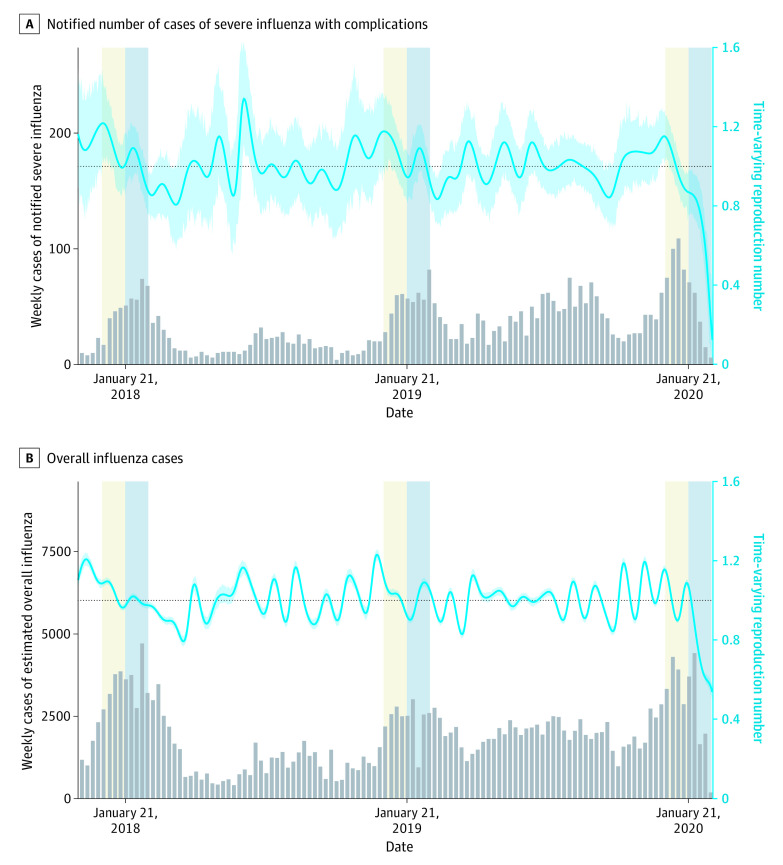
Incidence and Time-Varying Reproduction Number of Influenza in Taiwan, 2018 to 2020 A, Estimates from the notified number of cases of severe influenza with complications. B, Estimates from the overall influenza cases derived from the frequency of consulting physicians about influenza-like illness and the positive rate from laboratory testing for influenza. The gray bars represent the number of weekly incident cases and the blue curves represent the time-varying reduction number with 95% CIs in the shaded area. The thirty-day windows before and after January 21 of the 3 illustrated years are highlighted (yellow and blue background). January 21, 2020, was the date of the first confirmed SARS-CoV-2 infection in Taiwan.

After rerunning and fitting the transmission model to the observed size distribution of transmission clusters in Taiwan (eFigures 4-5 in the Supplement), we estimated that the R_p was 1.30 (95% CrI, 1.03-1.58), suggesting a 35%, 48%, and 57% reduction if the counterfactual R_0_ was 2.00, 2.50, and 3.00, respectively. We then compared the level of reduction from R_0_ to R_p with the reduction of time-varying reproduction number Rt of influenza before and after the COVID-19 epidemic. An early and sustained decline of influenza cases was found during the 2019 to 2020 season compared with the 2017 to 2018 and 2018 to 2019 seasons (Figure 3A; eTable 4 in the Supplement). The estimated Rt based on severe influenza in 2020 dropped from 0.87 on January 21, 2020 (when the first case COVID-19 was reported), to 0.27 1 month later, corresponding with a 69% decline. The analysis of estimated influenza of any severity showed a similar pattern, with a 47% Rt reduction from 1.07 on January 21, 2020, to 0.57 after January 21 (Figure 3B).

### Effectiveness of Joint Case-Based and Population-Based Interventions and Epidemic Projections

**Figure 4.**
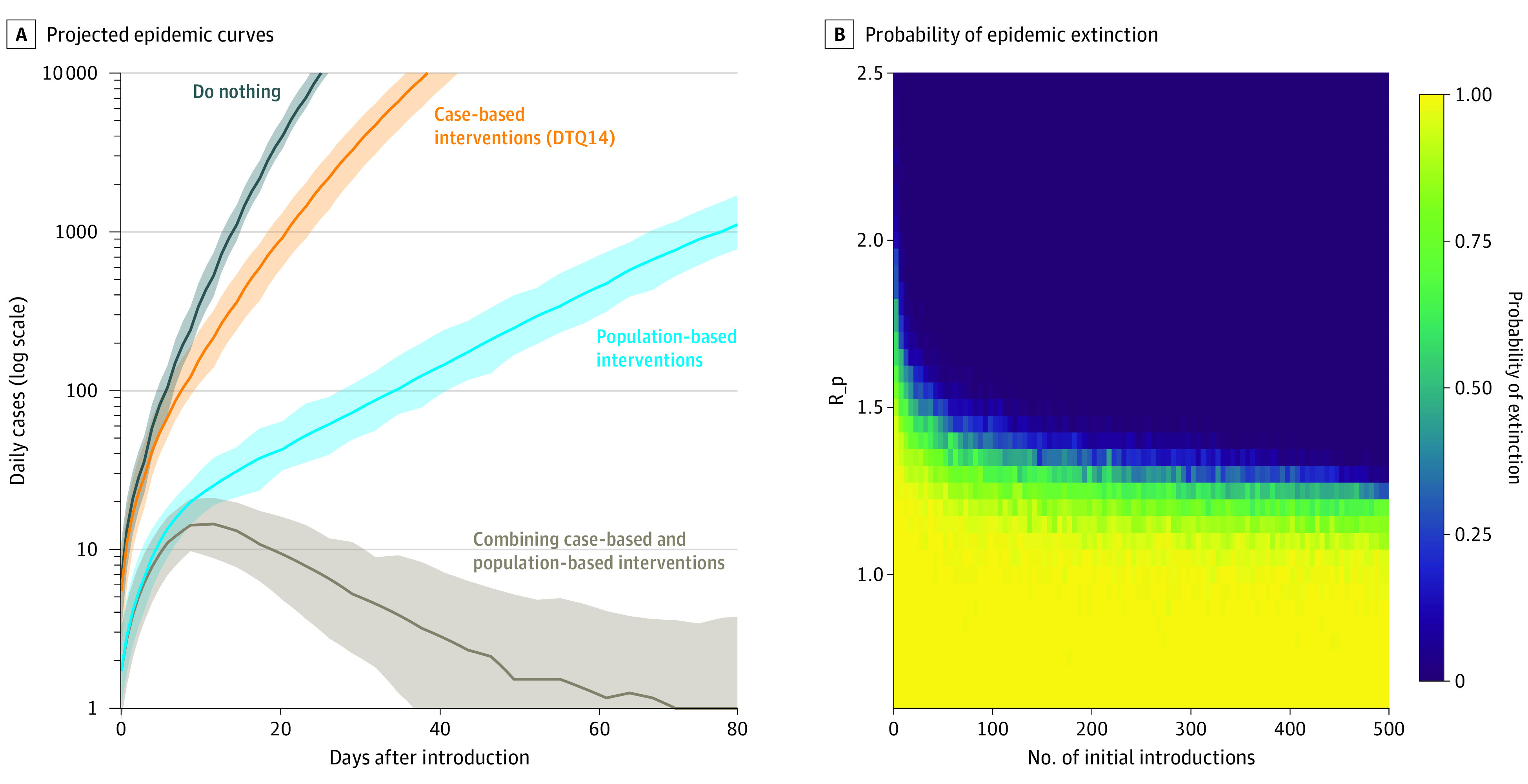
Projections on Epidemic Trajectory and Probability of Epidemic Extinction Under Joint Case-Based and Population-Based Interventions A, The projected epidemic curves with 100 initial introductions under different scenarios regarding the intervention being implemented. The 4 scenarios included (1) no intervention, (2) case-based interventions only, (3) population-based interventions only, and (4) combining case-based and population-based interventions. We assumed a sensitivity of 95% for case detection, an ascertainment probability for contact tracing of 90%, and a 48% reduction in background input basic reproduction number (R_0_) by population-based interventions (R_0_, 2.5; R_p, 1.3). The uncertainty intervals were calculated by the 2.5th and 97.5th percentiles from 1000 replicate simulations. B, The probability of epidemic extinction using case-based interventions (detection, contact tracing, and 14-day quarantine of close contacts) under different levels of population-based interventions (R_p) and initial numbers of introductions. Each cell presents the estimated probability of extinction based on 100 replicate simulations using the transmission model. D indicates case detection; T, contact tracing; Q14, quarantine of contacts for 14 days.

After fitting the transmission model to the size distribution of transmission clusters observed in Taiwan, the R_pc was estimated to be 0.85 (95% CrI, 0.78-0.89), while the R_pc that was estimated analytically from the average size of clusters was 0.62 (95% CrI, 0.45-0.72). We then projected the epidemic curve with 100 initial cases under different scenarios using the fitted model (R_0_, 2.50; R_c, 1.53; R_p, 1.30; R_pc, 0.85). Under the scenarios examined, we found that although case-based interventions and population-based interventions could each partially suppress the epidemic, exponential growth would continue if either category of interventions was used alone. By day 60, the daily number of new cases would rise to 37 631 (95% CrI, 29 586-46 285) and 481 (95% CrI, 320-736) for case-based and population-based interventions, respectively (Figure 4A). In contrast, combining case-based and population-based interventions would contain the epidemic; by day 60, the daily number of new cases would be 1.7 (95% CrI, 0.3-6.7) with the combined interventions (Figure 4A). By day 84 (95% CrI, 51-137), the daily number of new cases would be 0.

To understand how case-based and population-based interventions could work together, we estimated the probability of successful COVID-19 containment in Taiwan when case-based interventions were combined with different levels of population-based interventions (expressed as different values of R_p) under different numbers of initial introductions of COVID-19 cases (Figure 4B). When the number of initial infections was set at 100, R_p had to remain below 1.2 to achieve an extinction probability of at least 90%. If R_p was greater than 2.0, it would be impossible for the combined interventions to contain the outbreak (extinction probability: 0) even when the number of circulating infections was small.

## Discussion

Using a flexible modeling approach that incorporated multiple sources of primary data about COVID-19, we examined the effectiveness of case-based and population-based interventions in Taiwan by estimating the degree to which reproduction numbers for SARS-CoV-2 were reduced. We found that case-based interventions alone were insufficient to contain the epidemic, even in a country where the public health and health care systems were not overwhelmed and an efficient contact tracing program was in place. We also found that population-based interventions reduced the reproduction numbers for COVID-19 by nearly 50% and played an important role in containment. Nonetheless, only the combination of case-based and population-based interventions was sufficiently powerful to end the epidemic in Taiwan.

Previous modeling studies suggested the effectiveness of contact tracing with corresponding management (either quarantine or active monitoring) in settings and countries with well-functioning public health systems, such as Taiwan.^15,16,17^ However, our findings suggest that even in a well-prepared setting, contact tracing alone would fail to eliminate a COVID-19 epidemic if multiple introductions of the virus were likely. This discrepancy is driven primarily by the growing understanding of the role of presymptomatic transmission (nearly half of the transmission events might occur before an individual had symptoms) and the challenges for shortening the delay from the onset of symptoms to isolation of the individual with infection.^31^ The effectiveness of contact tracing depends on the timeliness of case detection and quarantine for high-risk contacts.^15,16^ According to our analysis of local data, the mean onset-to-isolation interval was about 5 days in Taiwan. This relatively long delay compared with the short serial interval (time between symptom onset in an infector-infectee pair) suggested that the virus had likely already been transmitted by the time of case notification and isolation. In our model, we assumed that testing and isolation occurred at the same time because it was the policy in Taiwan. However, in other settings, there are likely to be delays between testing, obtaining test results, and isolation; thus, the effects of case-based interventions would likely be further diminished.^16,32,33^ Our findings suggest that case-based interventions against COVID-19 should always be implemented along with effective population-based interventions, even in settings in which the case-based interventions are comprehensive. Moreover, our model found similar results for 7-day and 14-day quarantine of close contacts, suggesting that the quarantine period could be shortened and the burden on the public health system reduced. As of March 2021, several countries had implemented (ie, Singapore) or were considering (ie, the US and Thailand) a shorter quarantine period.^34,35,36^

We found that population-based interventions likely played an important role in the COVID-19 containment efforts in Taiwan. A meta-analysis of 172 observational studies in health care and non–health care settings found that physical distancing, face mask use, and eye protection were significantly associated with reduced COVID-19 transmission at the individual level.^37^ Empirical evidence about the population-level effects of behavioral changes has been limited. Most prior studies evaluated the change of time-varying reproduction number Rt as interventions were being implemented. In these studies, other concurrent interventions, including lockdown or contact tracing, were also in place, making it difficult to identify the independent effects of behavioral changes.^2,7^ Nonetheless, the persistent high burden of COVID-19 in countries with lower influenza activity in 2020 (such as the US, Australia, Chile, and South Africa) suggest that population-based interventions alone might not be sufficient to contain the epidemic.^38^

Our study provides a framework to consider the role of different sets of nonpharmaceutical interventions at different stages of the COVID-19 pandemic and in different settings. Border control may be an option to limit the epidemic at the early stage by reducing the number of introductions, especially in island nations like Taiwan and New Zealand.^6,9^ However, the effects of border closings could be quickly diminished when the number of introductions increases and the probability of local transmission increases correspondingly.^39,40^ When local transmission of SARS-CoV-2 occurs but the total number of cases remains low, our analysis suggests that elimination can still be achieved through the combination of intensive case-based and population-based interventions.

As the pandemic has evolved into widespread transmission (such as in the US and Europe), intensified contact tracing becomes logistically difficult; it is not sustainable when the public health system is overwhelmed. In this case, the effectiveness of conventional case-based interventions would be limited. A potential alternative is digital contact tracing through an electronic exposure notification system.^41^ As a result of substantial presymptomatic and asymptomatic transmission, a recent modeling study by the US Centers for Disease Control and Prevention found that identifying and isolating persons with symptomatic COVID-19 alone would prevent less than 50% of new infections.^42^ The US Centers for Disease Control and Prevention concluded that population-based measures and strategic testing of people without symptoms was essential for suppressing the pandemic before universal availability of vaccines. The US findings and our results suggest that in settings with generalized COVID-19 epidemics, priority should be given to population-based interventions over case-based and symptom-based strategies, and the intensity of population-based interventions should be increased to compensate for the decreased efficiency of case-based interventions. Nonetheless, maintaining behavioral changes, such as physical distancing and facial masking, can be challenging. “Pandemic fatigue” may lead to decreasing effects of population-based interventions.^43,44^ Finally, although the successful containment of the pandemic in Taiwan may not be replicated in countries with higher levels of transmission, our analysis suggests that it may still be possible to achieve suppression within these countries in specific settings where epidemic control efforts can be focused and intensified (eg, professional sports leagues and essential workplaces).^45^

### Limitations

Our study has limitations. First, as with all modeling studies, our results may be affected by the assumptions and input parameter values. Detailed case series data from contact tracing were used to inform the parameters and calibration; multiple sensitivity analyses explored the associations of varying the modeling assumptions with the conclusions. Second, the analysis was conducted in Taiwan, an island nation with the ability to control new case introductions through border control; therefore, our findings may not be generalizable to other settings. This is the reason that we focused on the effectiveness of case-based and population-based interventions on local transmission (instead of border control on the number of introductions). Third, we could not directly estimate the counterfactual R_0_ (ie, the hypothetical reproduction number without interventions) because most interventions were triggered quickly when COVID-19 was introduced.^8,9^ Nonetheless, our main conclusions were robust within the range of commonly reported R_0_ values of 2 to 3.^7,23,24^ Fourth, the analysis of cluster-size distribution relied on assuming complete information about all clusters. If small clusters (including isolated cases without further transmission) were more likely to be missed by surveillance and contact tracing, the estimated R_pc would have been lower, and the effects of population-based intervention would have been greater.

## Conclusions

This analysis suggests that population-based interventions are essential for controlling local transmission of SARS-CoV-2, even in settings with comprehensive contact tracing programs and effective public health systems. The combination of case-based interventions with population-based interventions with wide adherence may have led to successful COVID-19 control in Taiwan. Although vaccinations programs are ramping up toward widespread availability and coverage, the full benefits will only be realized over time. The experience in Taiwan suggests that mitigating the severity of the pandemic requires the collaborative effort of public health professionals and the general public.

## Notes

Supplement. eMethods. eFigure 1. Conceptual framework for the transmission model eFigure 2. Examples of the effects of case detection, contact tracing, and quarantine eFigure 3. Tornado diagrams from the one-way sensitivity analysis on the effects of case-based interventions eFigure 4. Model fitting to the observed serial intervals eFigure 5. Model fitting to the observed cluster sizes eFigure 6. The incidence and instantaneous reproduction number (Rt) of influenza in Taiwan, 2018–2020 eTable 1. The values of fixed parameters and the priors of fitted parameters in model fitting to the serial interval eTable 2. The values of fixed parameters and the priors of fitted parameters in model fitting to the cluster size distribution eTable 3. Scenarios in the assessment of case-based interventions eTable 4. The estimated time-varying reproduction number (Rt) of influenzae in Taiwan on January 21, February 4, and February 18, 2018–2020 eReferences
