# Supplementary material for: Comparison of Estimated Effectiveness of Case-Based and Population-Based Interventions on COVID-19 Containment in Taiwan
Source: JAMA Intern Med. 2021 Apr 6;181(7):913–21. doi: 10.1001/jamainternmed.2021.1644 (PMC8025126; doi:10.1001/jamainternmed.2021.1644)
Supplement: Supplement. — eMethods. eFigure 1. Conceptual framework for the transmission model eFigure 2. Examples of the effects of case detection, contact tracing, and quarantine eFigure 3. Tornado diagrams from the one-way sensitivity analysis on the effects of case-based interventions eFigure 4. Model fitting to the observed serial intervals eFigure 5. Model fitting to the observed cluster sizes eFigure 6. The incidence and instantaneous reproduction number (Rt) of influenza in Taiwan, 2018–2020 eTable 1. The values of fixed parameters and the priors of fitted parameters in model fitting to the serial interval eTable 2. The values of fixed parameters and the priors of fitted parameters in model fitting to the cluster size distribution eTable 3. Scenarios in the assessment of case-based interventions eTable 4. The estimated time-varying reproduction number (Rt) of influenzae in Taiwan on January 21, February 4, and February 18, 2018–2020 eReferences [file jamainternmed-e211644-s001.pdf]

Supplemental Online Content

Ng T-C, Cheng H-Y, Chang H-H, et al. Comparison of estimated effectiveness of case-based and population-based interventions on COVID-19 containment in Taiwan. *JAMA Intern Med*. Published online April 6, 2021. doi:10.1001/jamainternmed.2021.1644

eMethods.
eFigure 1. Conceptual framework for the transmission model
eFigure 2. Examples of the effects of case detection, contact tracing, and quarantine
eFigure 3. Tornado diagrams from the one-way sensitivity analysis on the effects of case-based interventions
eFigure 4. Model fitting to the observed serial intervals
eFigure 5. Model fitting to the observed cluster sizes
eFigure 6. The incidence and instantaneous reproduction number (R_t) of influenza in Taiwan, 2018–2020
eTable 1. The values of fixed parameters and the priors of fitted parameters in model fitting to the serial interval
eTable 2. The values of fixed parameters and the priors of fitted parameters in model fitting to the cluster size distribution
eTable 3. Scenarios in the assessment of case-based interventions
eTable 4. The estimated time-varying reproduction number (R_t) of influenzae in Taiwan on January 21, February 4, and February 18, 2018–2020
eReferences

This supplemental material has been provided by the authors to give readers additional information about their work.

© 2021 Ng T-C et al. JAMA Internal Medicine.

eMethods

1. Estimation of the interval parameters

The incubation period, the onset-to-isolation interval, and the serial interval (time interval between symptom onsets in an infector-infectee pair) were estimated from the dates (or date intervals if the exact date is unknown) of exposure, symptom onset, isolation of the confirmed SARS-CoV-2 cases in Taiwan. We estimated the distributions of these interval parameters using a Bayesian framework[1] that deals with the situation where the exact date is uncertain for both ends of the interval (i.e., doubly interval-censored). This method uses a hierarchical model to estimate the exact two ends of the time interval for each individual as well as the overall distribution of the interval among the population. In the case of incubation period estimation, the exact infection and onset times were unobserved and uniform priors bounded by individual exposure and onset windows were assumed. For each individual the exact time points of infection and symptom onset are modelled as follows.

$$X_i^{start} \sim Uniform\left(X_{l,i}^{start}, X_{u,i}^{start}\right)$$
$$X_i^{end} \sim Uniform\left(X_{l,i}^{end}, X_{u,i}^{end}\right)$$
$$Y_i = X_i^{end} - X_i^{start}$$

X_i^{start} is the exact time of infection, which was not observed. $\left[X_{l,i}^{start},\ X_{u,i}^{start}\right]$ represents the time window within which transmission could have occurred, and was obtained based on patients' travel/exposure history in case investigation reports. Likewise, X_i^{end} is the unobserved exact time of symptom onset and $\left[X_{l,i}^{end},\ X_{u,i}^{end}\right]$ is the time window within which symptom onset could occurred. Y_i is the individual incubation period, and was assumed to follow a gamma distribution with the shape (α) and the scale (β). We applied flat exponential priors to α and β as follows.

$$Y_i \sim Gamma(\alpha, \beta)$$
$$\alpha \sim Exponential(1/1{,}000)$$
$$\beta \sim Exponential(1/1{,}000)$$

To estimate the serial interval, we simply replaced the exposure-to-onset quantities with onset-to-onset quantities between the primary and secondary cases. To estimate the onset-to-isolation interval, we simply replaced the exposure-to-onset quantities with onset-to-isolation quantities Specifically, we added an offset of 1 days for both the serial interval and the onset-to-isolation interval (i.e. add 1 days on each interval $Y\ \ i$) because there are negative values generated by the observed windows. We included these observations for that negative serial intervals and negative onset-to-isolation intervals are plausible in practice. Particularly, negative onset-to-isolation intervals occurred due to contact tracing and active surveillance measures, and negative serial intervals occurred when the incubation period of the index case happened to be much longer than the incubation period of the secondary case.

For each estimation, we ran 4 Markov chain Monte Carlo (MCMC) chains using the Non-U-Turn Sampler in Stan.[2] Each chain contains 1,000 warm-up iterations and 500 samples, rendering to a total of 2,000 samples. The credible intervals are obtained from the 2.5th and 97.5th percentiles in the posterior predictive simulations of the gamma distributions.

2. The dynamical transmission model

2.1 Overview

We adopted the structure of the branching process model developed by Hellewell, et al.,[3] which in essence consists of two components: 1) the branching process, and 2) the intervention model. The branching process simulates the disease transmission dynamics in the early phase of an outbreak. The intervention model considers the effects of various case-based interventions for COVID-19 control. Specifically, case detection, contact tracing, and quarantine

© 2021 Ng T-C et al. JAMA Internal Medicine.

of close contacts are considered. The parameters and their values for this dynamical transmission model are listed in Table 1.

2.2 The branching process

The branching process simulates growing transmission trees starting from some given initial cases. This process is implemented by the following steps.

i. Initiate with initial active cases.
ii. For each active symptomatic case, determine the onset time and the testing time by drawing an individual incubation period and an onset-to-isolation interval from their probability distributions (estimated from case series data). The testing time for active asymptomatic cases are set to infinite.
iii. For each active case (both symptomatic and asymptomatic), draw the number of newly infected cases from the distribution of secondary cases.
iv. For each active case (both symptomatic and asymptomatic), apply the intervention model (see eMethods 2.3 for more details) and determine the period of quarantine and isolation.
v. For each secondary case, determine the infection time by drawing an individual generation interval (infection times in an infector-infectee pair).
vi. For each transmission pair, determine whether the transmission is realized, or prevented by comparing the infection time of the secondary case and the period of quarantine/ isolation of the index case.
vii. Deactivate the index cases, and activate the realized secondary cases.
viii. Repeat step ii~vii, until there is no active case, or when the maximal number of generations is reached.

The distributions of the incubation period and onset-to-isolation interval were estimated from case series data with the method described in eMethods 1. We assumed a negative binomial distribution for the secondary case distribution, which is governed by the reproduction number (R) and the dispersion parameter (k). We assume that $R = R_0 = 2.5$ in the counterfactual scenario where no interventions and behavioral changes were in effect. In the real-world setting R should equal R_p, denoting for the reproduction number under the effect of population-based interventions. R_p and k were estimated by fitting the dynamical model to the observed cluster sizes (see eMethods 3.3). The generation interval distribution was assumed to be a skewed normal distribution centered at each index case's onset time to avoid the inconsistent length of incubation periods and generation intervals. This parameterization also makes the proportion of pre-symptomatic transmission (p^{pre}) an explicit parameter in our model. The standard deviation of the generation interval (σ), and the proportion of pre-symptomatic transmission (p^{pre}) were estimated by fitting the dynamical model to the observed serial intervals (see eMethods 3.2). Regarding the possibility of asymptomatic infection, we assumed a fixed probability of being asymptomatic ($p^{asym} = 0.4$), and fixed relative transmissibility ($r^{asym} = 0.5$) for all infections.

2.3 The intervention model

The intervention model is composed of a set of rules which determine whether and when active cases and their contacts are quarantined or isolated and hence unable to transmit disease. Specifically, case detection, contact tracing, and quarantine of close contacts were implemented as described in the following.

i. Case detection: Each active, untraced, and symptomatic case was tested with probability θ, and was immediately isolated if tested positive. The secondary cases generated during the incubation period (pre-symptomatic period) and the onset-to-isolation interval cannot be prevented.
ii. Contact tracing: Each secondary case (except for initial introductions) was ascertained as a close contact (successfully traced) of the detected index case with probability ρ. If a secondary case was ascertained and showed symptoms on the time of contact tracing (the onset time of the detected index case), the secondary case was immediately isolated, unless the case had already been detected and isolated. Case detection plus contact tracing was able to prevent the transmission during the onset-to-isolation interval, but not during the incubation period.
iii. Quarantine of close contacts: Each active and traced case (regardless of the presence of symptoms) was immediately quarantined at the time of being traced. If the case develops symptoms during the quarantine

© 2021 Ng T-C et al. JAMA Internal Medicine.

period, he/she will be immediately isolated. Only the combination of detection, tracing, and quarantine was able to prevent pre-symptomatic transmissions and transmissions from asymptomatic cases.

Note that asymptomatic cases were never detected, traced or isolated, but could be quarantined. We also assumed perfect compliance with isolation and quarantine order, and all transmissions were prevented during that period. eFigure 2 gives examples that illustrate the effects of these interventions on the prevention of disease transmission. eFigure 3 provides the one-way sensitivity analysis of all the parameters in this model.

3. Parameter estimation and model calibration

3.1 Overview and the Sequential Monte Carlo (SMC) algorithm

The observed serial intervals and size distribution of stuttered transmission chains were utilized to calibrate the dynamical model and to estimated related parameters. We note that the reproduction number (R) and the dispersion parameter (k) only affect the number of infections, not the temporal relationship between successive generations, e.g. the distribution of the serial interval. In contrast, the probability of pre-symptomatic transmission (p^{pre}) and the standard deviation of the generation interval (σ) affect the number of secondary infections because they interact with case-based interventions, such that pre-symptomatic transmissions are hardly prevented by case-based interventions. Therefore, we first calibrated the model and estimated p^{pre} and σ using the observed serial intervals. Then the resulting model was used for further calibration to the observed cluster sizes, and for the estimation of R_p and R_{pc}.

Since the likelihood of the dynamical model is intractable, we used a sequential Monte Carlo algorithm to obtain posterior distributions of parameters of interest. This algorithm was used to fit branching process-based dynamical models in a similar context.[4,5]. The algorithm started from a population of 1,000 parameter sets drawn from the prior distributions. Data were simulated with the branching process model parameterized by these parameter sets, and the distance between the simulated and empirical data was measured by Kolmogorov–Smirnov (*KS*) statistics. In each round of iteration, the parameter set was resampled, perturbed, and passed on until the criteria of convergence were met. We ran 4 chains of SMC algorithms to generate a total of 4,000 posterior samples for inference.

The steps of an SMC algorithm are as follows:

i. Initiation: generate a population of 1,000 parameter sets by Latin Hypercube sampling from prior distributions.
ii. Simulation: simulate data points with each parameter set and the dynamical model.
iii. Evaluation: calculate the *KS* statistics.
iv. Evolution: resample a new population of 1,000 parameter sets from the current population weighted by $1/KS^2$.
v. Mutation: perturb each new parameter set by up to 10%.
vi. Repeat step ii~v until the median *KS* statistics of the population is less than 0.05 and is within 10% of each of the previous two rounds.

3.2 Model calibration with observed serial intervals

The probability of pre-symptomatic transmission (p^{pre}) and the standard deviation of the generation interval (σ) were estimated by fitting the model to the observed serial interval distribution. Wide uniform priors were assigned to both parameters. The posterior mean estimates are 0.55 (95%CrI 0.41-0.68) and 2.70 (95%CrI 1.88-3.76), for $p^{pre}$and σ, respectively. eTable 1 lists the values of priors, posterior estimates and other fixed parameters used in this calibrating stage. eFigure 4 presents the posterior distributions, the convergence plots, and the posterior predictive of the serial interval.

© 2021 Ng T-C et al. JAMA Internal Medicine.

3.3 Model calibration with observed cluster sizes

The reproduction number under population-based interventions only (R_p), and the dispersion parameter (k) were estimated in this stage, by fitting the model to observed cluster sizes. Wide uniform priors were assigned to the parameters. The posterior mean estimates are 1.30 (95%CrI 1.03-1.58) and 29.21 (95%CrI 6.28, 50.00), for $R_p$and k, respectively. eTable 2 lists the values of priors, posterior estimates and other fixed parameters used in this calibrating stage. eFigure 5 presents the posterior distributions, the convergence plots, and the posterior predictive of the cluster sizes. The limited amount of observations for cluster sizes make it hard to infer the dispersion parameter, as the posterior distribution of $k$did not converge well (eFigure 5B). However, the posterior distribution of R_p did converge to consistent estimates (eFigure 5A) which had accounted for the uncertain in k simultaneously.

4. Estimation of the time-varying reproduction numbers

The time-varying reproductive number (R_t) for SARS-CoV-2 and influenza were estimated using Wallinga and Teunis method,[6] also known as the "case reproduction number".[7] This method attributes the transmission events and assigns the value of R_t to the cohort of primary cases at time t. Since R_t represents the transmissibility of primary cases, it explains the future incidence, and can reflect the subsequent impacts of events after specific time points.[8]. Practically, this method estimates the transmission probabilities between every possible transmission pair, according to their observed serial intervals. The probability that case i was infected by case j (p_{ij}) is given as

$$p_{ij} = \frac{w(t_i - t_j)}{\sum_{m \neq i} w(t_i - t_m)}$$

p_{ij} is calculated by normalizing the likelihood of case j infecting case i by the sum of the likelihood from all possible infector cases $m \neq i$. $w(t_i - t_j)$ is the transmission likelihood quantifying how well the observed serial interval (the onset time difference between case i and j, $t_i - t_j$) fits the serial interval distribution of ascertained transmission pairs. The effective reproduction number of case j is by definition (the expected number of secondary infections) the sum of all the transmission probabilities where case j is the infector.

$$R_j = \sum_i p_{ij}$$

We then summarized the R_j's into the time-varying reproduction numbers (R_t) by calculating the 7-day moving averages according to their onset time. The confidence intervals were calculated by the 2.5th and 97.5th percentiles in the R_t of 100 simulated transmission trees from the p_{ij}'s matrix, as in Cori, et al.[5]

For SARS-CoV-2, we directly used the daily incidence based on the symptom onset date to estimate R_t. For influenzae, the weekly incidence from two different data sources were used, including the notified influenzae cases with severe complications in the National Notifiable Disease Surveillance System, and the influenzaee-like illness (ILI) consulting rate in the out-patient and emergency departments. The ILI consultation rate was further multiplied by the positive rate of influenzae according to the laboratory surveillance data. Cubic spline smoothing was used to disaggregate the weekly-basis data into daily-basis incidence.[9] To avoid the problem of right truncation, we used the influenzae data through the end of March to estimate the Rt until Feb 21st (one month after the first reported case of Covid-19 in Taiwan). Another key input to R_t estimation is the distribution of the serial interval. For SARS-CoV-2, the serial interval was estimated using the ascertained transmission pairs in our case series data. For seasonal influenzae, we assumed the mean and standard deviation of the serial interval to be 3.6 and 1.6 according to a previous study[10]. In addition to the Wallinga and Teunis method, we also estimated the instantaneous (real-time) reproduction number *Rt* by Cori et al. for the influenzae analysis, and the results were shown in eFigure 6.

© 2021 Ng T-C et al. JAMA Internal Medicine.

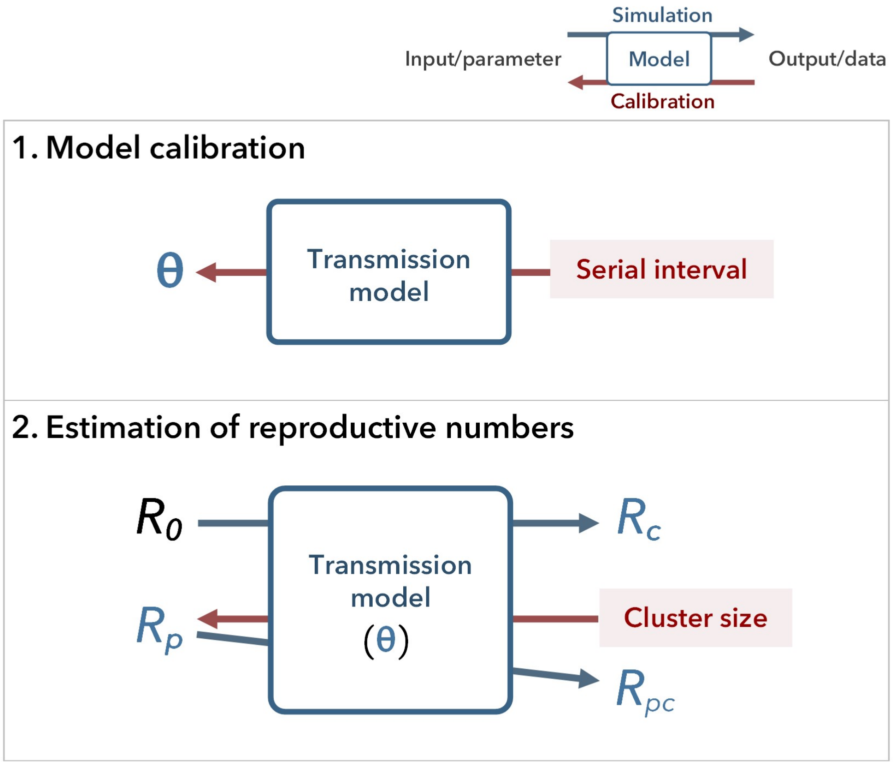

eFigure 1. Conceptual framework for the transmission model.

The transmission model is a stochastic branching process model that simulates the epidemic based on given input parameters (in a forward fashion). On the other hand, the transmission model could be used to estimate the parameter values through fitting/calibrating the model output to the observed data (in a backward fashion). A two-step approach was used in this study. First, the stochastic model was calibrated to the observed serial interval distribution to estimate the proportion of pre-symptomatic transmission and the standard deviation of generation interval (eMethods 3.2). Second, the calibrated model from the first step was used to estimate and project the potential effectiveness of case-based interventions in the absence of population-based interventions (the arrow from R_0 to R_c). In this case, R_0 is the input parameter and R_c is the output from the model. The model was also used to estimate potential effectiveness of population-based interventions and joint interventions (the arrow from R_p to R_{pc}). In this case, R_p is the (unknown) input parameter and R_{pc} is the output from the model that depended on R_p. By fitting the cluster size distribution from the model to the cluster size distribution observed in contact tracing, R_p and R_{pc} were jointly estimated.

R_0, basic reproduction number (assumed); R_c, effective reproduction number with case-based interventions (estimated); R_p: effective reproduction number with population-based interventions (estimated); R_{pc}, effective reproduction number with both case-based and population-based interventions.

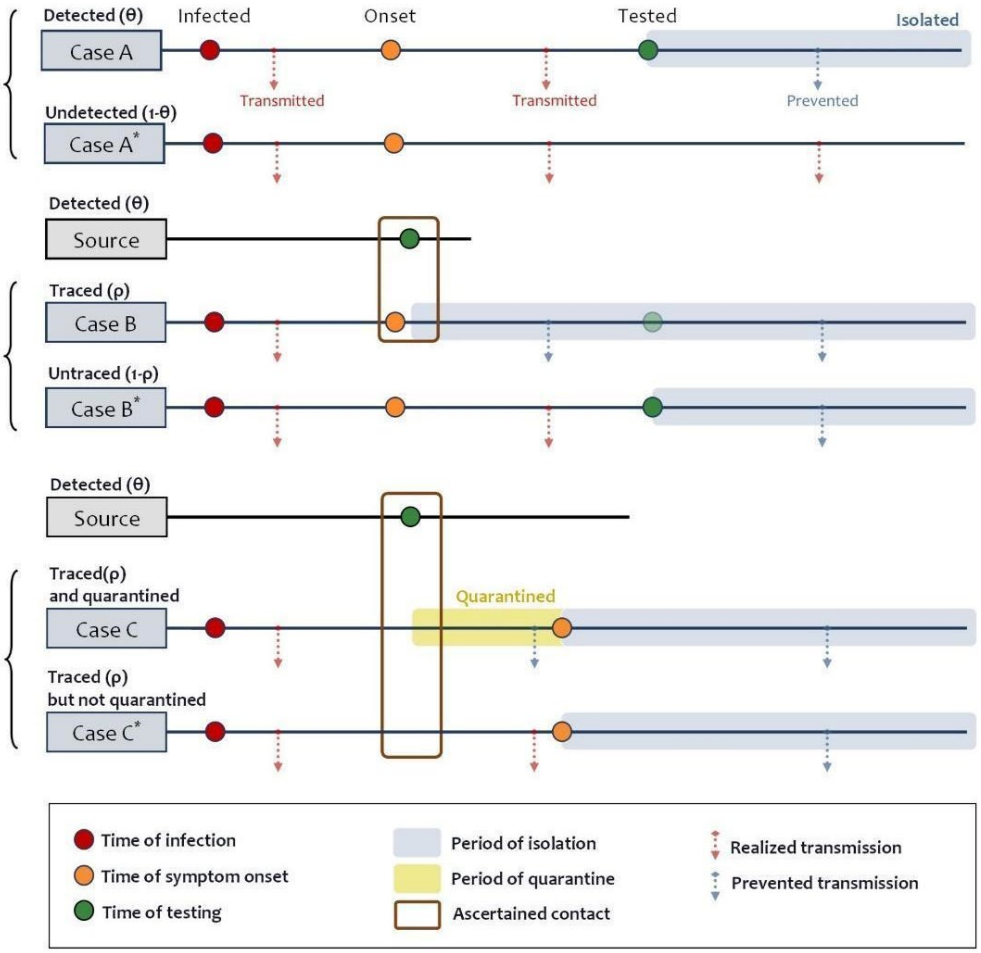

eFigure 2. Examples of the effects of case detection, contact tracing, and quarantine.

Case A and A* demonstrate the effect of mere detection, which can only prevent the transmission once the active cases are tested and isolated. That is, the active cases can transmit the disease during their incubation and delay of case detection. Case B and B* demonstrate the effect of detection plus tracing (without quarantine), where case B was successfully traced and onset within a buffer period. Therefore, case B was immediately isolated when the source was detected. Detection plus tracing can prevent transmission during the delay of case detection. Case C and C* demonstrate the combined effect of detection, contact tracing, and quarantine. Only in this scenario that transmission during the incubation period can be prevented. We assume that there is no delay between testing and isolation, the buffer of contact tracing to be one day, and the same effect of quarantine and isolation. Besides, asymptomatic cases are never detected or traced but could be quarantined and have lower transmissibility.

© 2021 Ng T-C et al. JAMA Internal Medicine.

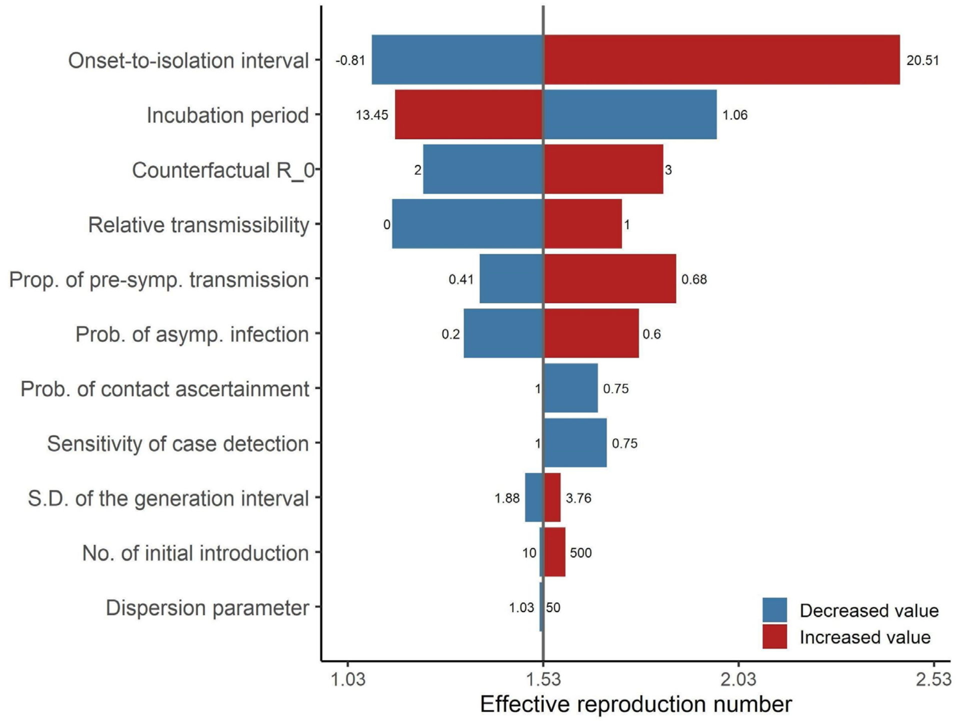

eFigure 3. Tornado diagrams from the one-way sensitivity analysis on the effects of case-based interventions.

The effective reproduction number under case-based interventions (R_c). The blue bars represent the change in the measured outcome when the corresponding parameter value decreased; the red bars represent the change when the parameter value increased. The tuning ranges of the parameters are shown next to ends of the bars.

© 2021 Ng T-C et al. JAMA Internal Medicine.

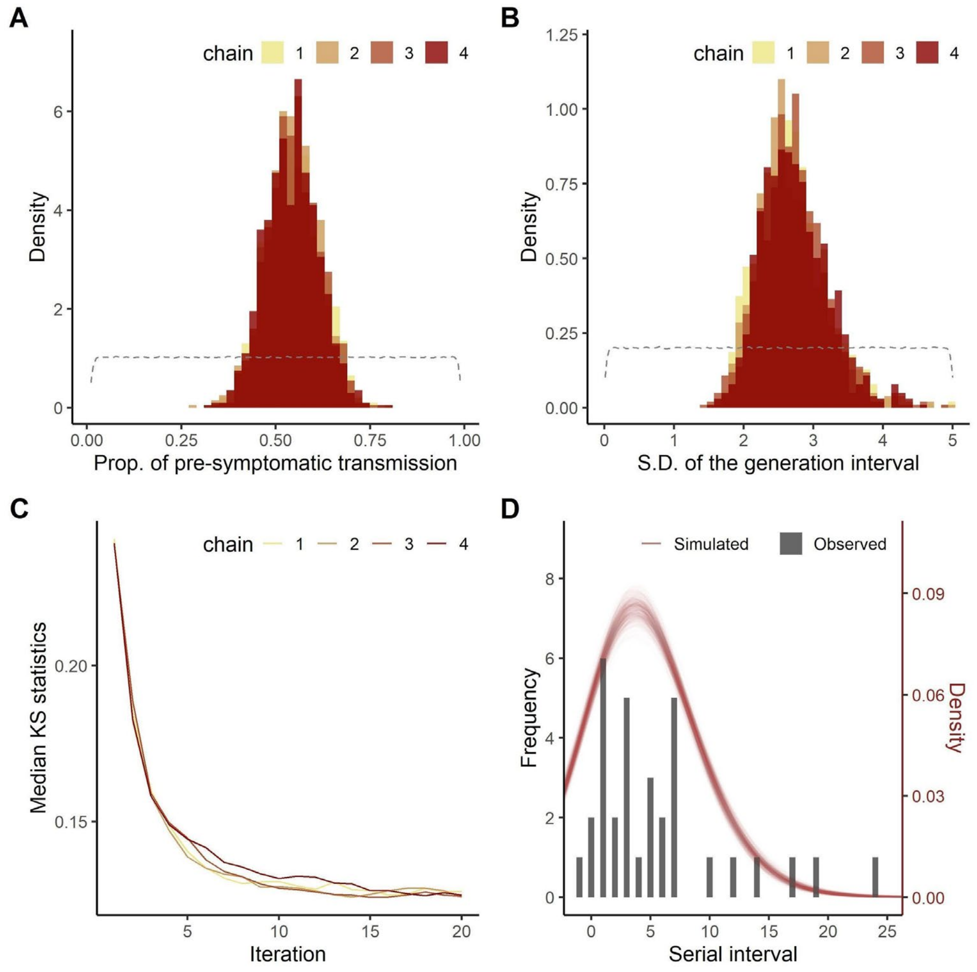

eFigure 4. Model fitting to the observed serial intervals.

(A) The posterior distribution of the proportion of pre-symptomatic transmission (p^{pre}). (B) The posterior distribution of the standard deviation of the generation interval (σ). (C) The convergence plot of the sequential Monte Carlo algorithm, with the median Kolmogorov–Smirnov (*KS*) statistics as the distance measure. (D) The distribution of observed serial intervals (grey bar) and the posterior predictive simulation of the serial interval distribution (100 times of simulation).

© 2021 Ng T-C et al. JAMA Internal Medicine.

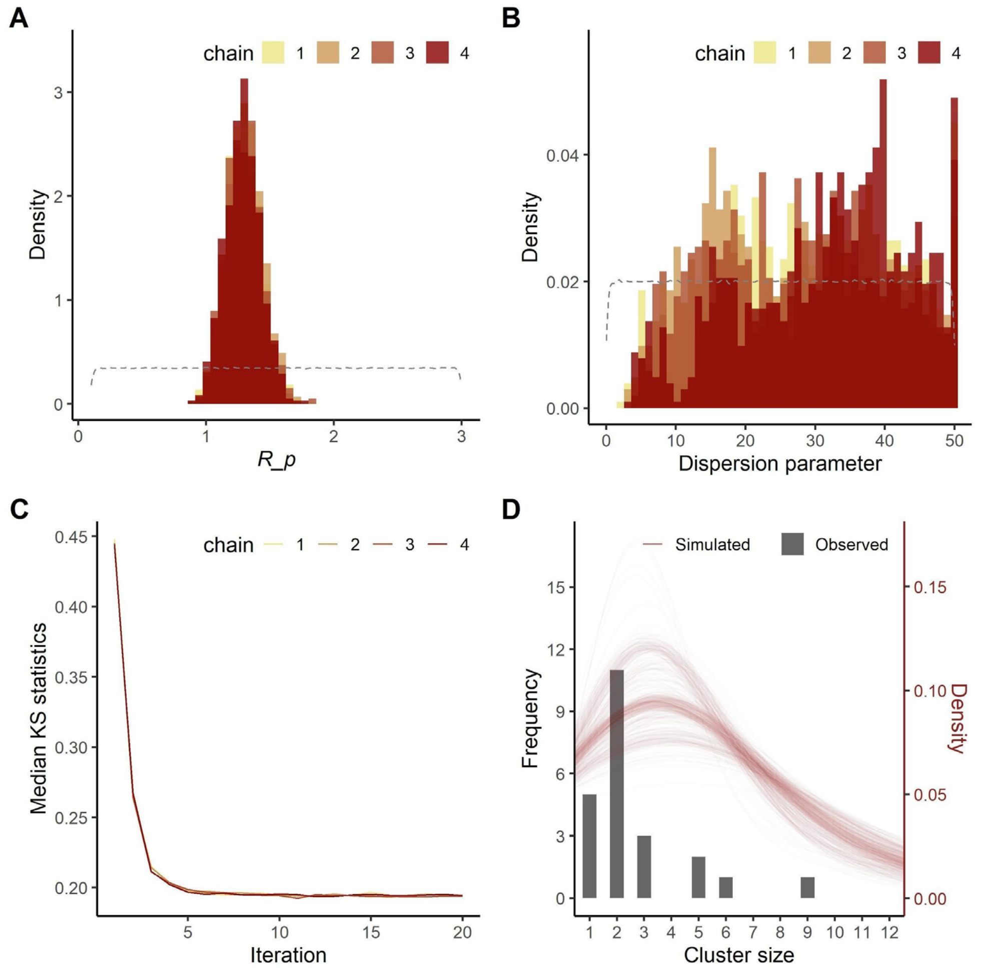

eFigure 5. Model fitting to the observed cluster sizes.

(A) The posterior distribution of the effective reproduction number under population-based interventions (R_p). (B) The posterior distribution of the dispersion parameter (k). (C) The convergence plot of the sequential Monte Carlo algorithm, with the median Kolmogorov–Smirnov (*KS*) statistics as the distance measure. (D) The observed cluster sizes and the posterior predictive simulation of the cluster size distribution (100 times of simulation).

© 2021 Ng T-C et al. JAMA Internal Medicine.

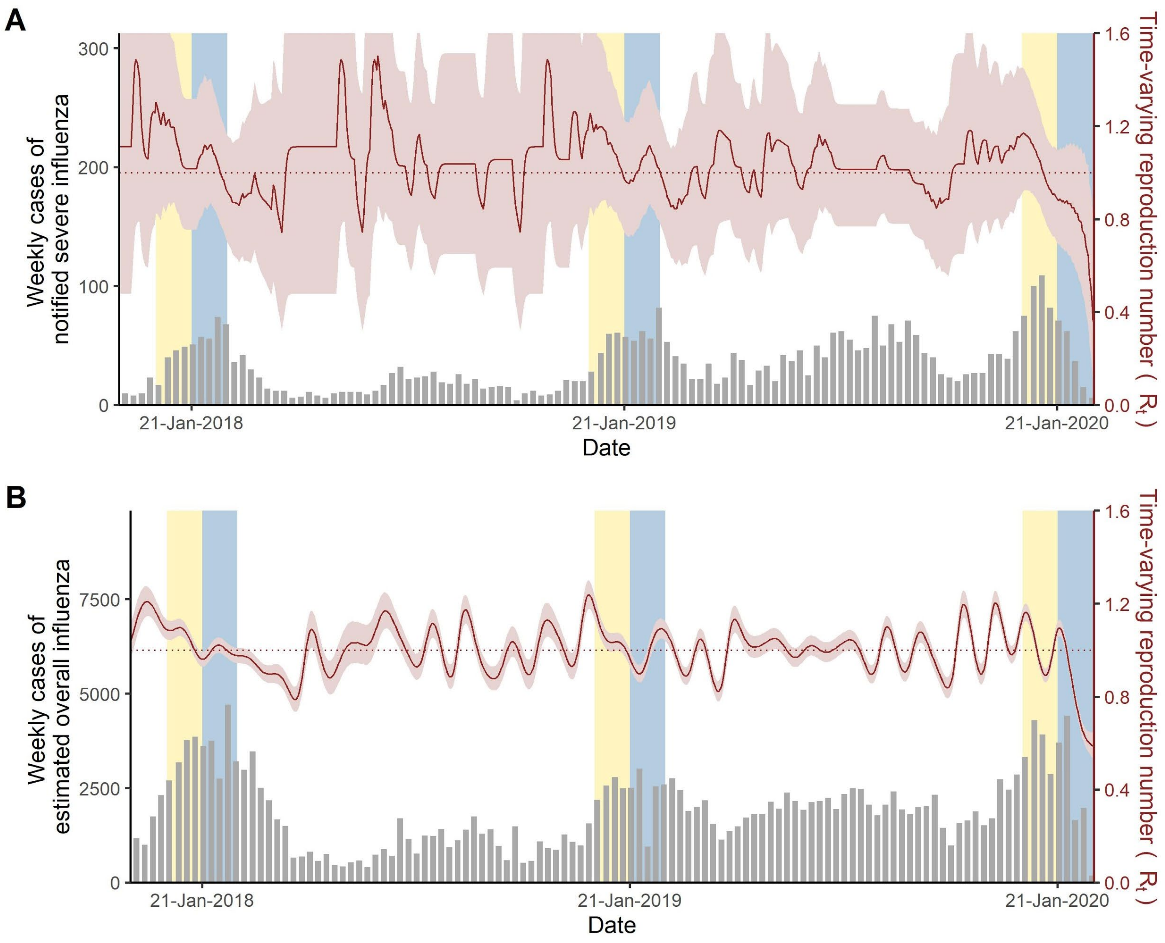

eFigure 6. The incidence and instantaneous reproduction number (Rt) of influenzae in Taiwan, 2018–2020.

(A) Estimates from the notified number of severe influenzae with complications. (B) Estimates from the overall influenzae cases derived from influenzae-like illness consultation rate and the positive rate from laboratory testing for influenzae (eMethods). The gray bars represent the number of weekly incidence cases, and the red lines represent the Rt with 95% confidence intervals in the shaded area. Thirty days before and after (yellow and blue background) January 21, the date of the first SARS-CoV-2 infection confirmed in Taiwan, was highlighted.

© 2021 Ng T-C et al. JAMA Internal Medicine.

eTable 1. The values of fixed parameters and the priors of fitted parameters in model fitting to the serial interval.

Parameter	Fixed value/ prior range	Posterior mean (95% CrI)
Mean incubation period, days	5.50	
Standard deviation of incubation period, days	3.26	
Mean onset-to-isolation interval, days	5.02	
Standard deviation of onset-to-isolation interval, days	5.80	
Basic reproduction number, R_0	2.5	
Dispersion parameter, k	20	
Probability of asymptomatic infection, p^{asym}	0,4	
Relative transmissibility of asymptomatic case, r^{asym}	0.5	
Proportion of pre-symptomatic transmission, p^{pre}	Uniform (0.01–0.99)	0.55 (0.41–0.68)
Standard deviation of the generation interval, σ	Uniform (0.001–5)	2.70 (1.88–3.76)
Probability of case detection, θ	0.95	
Probability of contact ascertainment, ρ	0.90	
Duration of quarantine, days	14	
Backtracking days for quarantined contacts	4	

© 2021 Ng T-C et al. JAMA Internal Medicine.

eTable 2. The values of fixed parameters and the priors of fitted parameters in model fitting to the cluster size distribution

Parameter	Fixed value/ prior range	Posterior mean (95% Crl)
Mean incubation period, days	5.50	
Standard deviation of incubation period, days	3.26	
Mean onset-to-isolation interval, days	5.02	
Standard deviation of onset-to-isolation interval, days	5.80	
Effective reproduction number, R_p	Uniform (0.1–3.0)	1.30 (1.03–1.58)
Dispersion parameter, k	Uniform (0.001–50)	29.21 (6.28–50)
Probability of asymptomatic infection, p^{asym}	0.4	
Relative transmissibility of asymptomatic case, r^{asym}	0.5	
Proportion of pre-symptomatic transmission, p^{pre}	0.55	
Standard deviation of the generation interval, σ	2.70	
Probability of case detection, θ	0.95	
Probability of contact ascertainment, ρ	0.90	
Duration of quarantine, days	14	
Backtracking days for quarantined contacts	4	

© 2021 Ng T-C et al. JAMA Internal Medicine.

eTable 3. Scenarios in the assessment of case-based interventions.

**Scenario**	**Probability of detection (θ)**	**Probability of contact ascertainment (ρ)**	**Duration of quarantine, days**
No intervention	0	0	0
Detection	0.95	0	0
Detection + Tracing	0.95	0.9	0
Detection + Tracing + 7-day Quarantine	0.95	0.9	7
Detection + Tracing + 14-day Quarantine	0.95	0.9	14

© 2021 Ng T-C et al. JAMA Internal Medicine.

eTable 4. The estimated time-varying reproduction number (R_t) of influenzae in Taiwan on January 21, February 4, and February 18, 2018–2020.

	**Notified severe influenzae**			**Estimated total influenzae**		
**Year**	**January 21**	**February 4**	**February 18**	**January 21**	**February 4**	**February 18**
2018	1.01 (0.88–1.15)	1.08 (0.96–1.21)	0.89 (0.78–0.98)	0.97 (0.96–0.99)	1.01 (1–1.03)	0.98 (0.96–0.99)
2019	0.95 (0.85–1.08)	1.08 (0.95–1.21)	0.95 (0.84–1.05)	0.92 (0.91–0.94)	1.01 (0.99–1.04)	1.07 (1.05–1.09)
2020	0.87 (0.76–0.97)	0.78 (0.67–0.94)	0.27 (0.12–0.53)	1.07 (1.06–1.09)	0.72 (0.71–0.74)	0.57 (0.54–0.60)

The daily incidence of influenzae was derived from the notified influenzae patients with severe complications or an estimated overall number of medical visits cases due to influenzae-like illness.

© 2021 Ng T-C et al. JAMA Internal Medicine.

eReferences

1. Backer JA, Klinkenberg D, Wallinga J. Incubation period of 2019 novel coronavirus (2019-nCoV) infections among travellers from Wuhan, China, 20–28 January 2020. *Eurosurveillance*. 2020;25(5):2000062. doi:10.2807/1560-7917.es.2020.25.5.2000062

2. Stan Development Team. 2020. Stan Modeling Language Users Guide and Reference Manual, 2.25. https://mc-stan.org

3. Hellewell J, Abbott S, Gimma A, et al. Feasibility of controlling COVID-19 outbreaks by isolation of cases and contacts. *Lancet Global Heal*. 2020;8(4):e488-e496. doi:10.1016/s2214-109x(20)30074-7

4. Peak CM, Childs LM, Grad YH, Buckee CO. Comparing nonpharmaceutical interventions for containing emerging epidemics. *Proc National Acad Sci*. 2017;114(15):4023-4028. doi:10.1073/pnas.1616438114

5. Peak CM, Kahn R, Grad YH, et al. Individual quarantine versus active monitoring of contacts for the mitigation of COVID-19: a modelling study. *Lancet Infect Dis*. 2020;20(9):1025-1033. doi:10.1016/s1473-3099(20)30361-3

6. Wallinga J, Teunis P. Different Epidemic Curves for Severe Acute Respiratory Syndrome Reveal Similar Impacts of Control Measures. *Am J Epidemiol*. 2004;160(6):509-516. doi:10.1093/aje/kwh255

7. Cori A, Ferguson NM, Fraser C, Cauchemez S. A new framework and software to estimate time-varying reproduction numbers during epidemics. *Am J Epidemiol*. 2013;178(9):1505-1512. doi:10.1093/aje/kwt133

8. Gostic KM, McGough L, Baskerville E, et al. Practical considerations for measuring the effective reproductive number, Rt. *Medrxiv*. Published online 2020:2020.06.18.20134858. doi:10.1101/2020.06.18.20134858

9. Cowling BJ, Ali ST, Ng TWY, et al. Impact assessment of non-pharmaceutical interventions against coronavirus disease 2019 and influenza in Hong Kong: an observational study. Lancet Public Heal. 2020;5(5):e279-e288. doi:10.1016/s2468-2667(20)30090-6

10. Cowling BJ, Fang VJ, Riley S, Peiris JSM, Leung GM. Estimation of the serial interval of influenza. *Epidemiology*. 2009;20(3):344-347. doi:10.1097/ede.0b013e31819d1092

© 2021 Ng T-C et al. JAMA Internal Medicine.
